# Case Report: Trans-Articular External Skeletal Fixation of the Hip for a Highly Comminuted Juxta-Articular Fracture of the Proximal Femur Caused by Gunshot Injury in a Cat

**DOI:** 10.3389/fvets.2021.652847

**Published:** 2021-07-19

**Authors:** Hyun-Jung Han, Kyeongpung Lee, Hun-Young Yoon

**Affiliations:** ^1^Department of Veterinary Emergency and Critical Care, Konkuk Veterinary Medical Teaching Hospital, Konkuk University, Seoul, South Korea; ^2^Department of Materials Science and Engineering, Seoul National University, Seoul, South Korea; ^3^Department of Veterinary Surgery, College of Veterinary Medicine, Konkuk University, Seoul, South Korea

**Keywords:** cat, comminuted fracture, external skeletal fixation, gunshot, juxta-articular, proximal femur, trans-articular, coxofemoral joint

## Abstract

This report describes a novel surgical technique for trans-articular external skeletal fixation (TA-ESF) of the hip to stabilize a rare, highly comminuted juxta-articular fracture of the proximal femoral segment involving the metaphysis and diaphysis in a cat. A 2-year-old, castrated male, Korean shorthair cat was admitted for a left femoral fracture caused by gunshot injury. Radiographs and computed tomography (CT) scans revealed a highly comminuted proximal femoral diaphyseal fracture that involved the femoral neck and the greater trochanter. The left femoral head was intact on CT. Under fluoroscopic guidance, an intramedullary Steinmann pin was placed to align the femur, and five positive-profile, end-threaded pins were placed in the left hemipelvis and distal femur. A triangulated frame of connecting bars was constructed using Steinmann pins and epoxy resin for TA-ESF. This secured the pins placed in the pelvis and femur as well as the intramedullary pin, providing proximal femoral stabilization by trans-articular fixation of the hip. The cat began placing weight on the left pelvic limb 4 days postoperatively, and progressively obtained near-normal limb function by day 112. The TA-ESF was partially dismantled to a linear tie-in construct on day 64 and was completely removed on day 161. Final radiographs on day 161 revealed lack of bone healing in the proximal segment, especially femoral head and neck, despite functional recovery of the injured leg. At one year postoperatively, the cat had normal limb function without any noticeable complications. On follow-up 29 months after surgery, the owner reported that the cat had normal limb function without any noticeable complications. Despite insufficient bone healing in the cat, TA-ESF of the hip allowed for satisfactory functional recovery of this challenging juxta-articular fracture of the proximal femur, which was not amenable to stabilization with a traditional non-load-sharing fixation system.

## Introduction

Surgical stabilization of highly comminuted fractures of the proximal or distal juxta-articular bones has rarely been reported in veterinary literature. These comminuted juxta-articular fractures are difficult to stabilize since the presence of a short proximal or distal segment limits the types of fixation that can be used (1). A circular external skeletal fixation (ESF) is preferred for fractures with very short proximal or distal segments in dogs and cats, particularly if the fracture cannot be reconstructed (1–3). The ring components of these ESFs, which use fixation wires, are particularly advantageous in such comminuted fractures with short segments since they provide the ability to use multiple points of fixation in extremely small fracture segments (1). However, the application of such circular ESFs is limited by the anatomical location. In the cat described in the present case, application of ring components of the circular ESF to the proximal femur was limited due to the proximity to the body wall (1).

This report describes a novel surgical technique for the successful management and functional recovery of a highly comminuted juxta-articular fracture of the proximal femur caused by a gunshot injury using trans-articular ESF (TA-ESF) of the hip in a cat. These results were obtained despite a lack of bone healing.

## Case Description

A 2-year-old castrated, male, Korean shorthair cat weighing 4.4 kg was admitted with a highly comminuted proximal femoral fracture caused by a gunshot injury sustained 3 days earlier. On presentation, the cat could not bear weight on the left pelvic limb. Additionally, there was a small skin puncture wound, suspected to be the bullet trajectory, over the left hemipelvis. The cat showed signs of pain during palpation of the left pelvis and pelvic limb, but did not show any neurological deficits. Abnormalities on a complete blood count and biochemistry panel included a decreased packed cell volume of 24.7% (reference range, 30.3–52.3%) and an elevated creatine kinase level of 675 U/L (reference range, 0–314 U/L).

**Figure 1:**
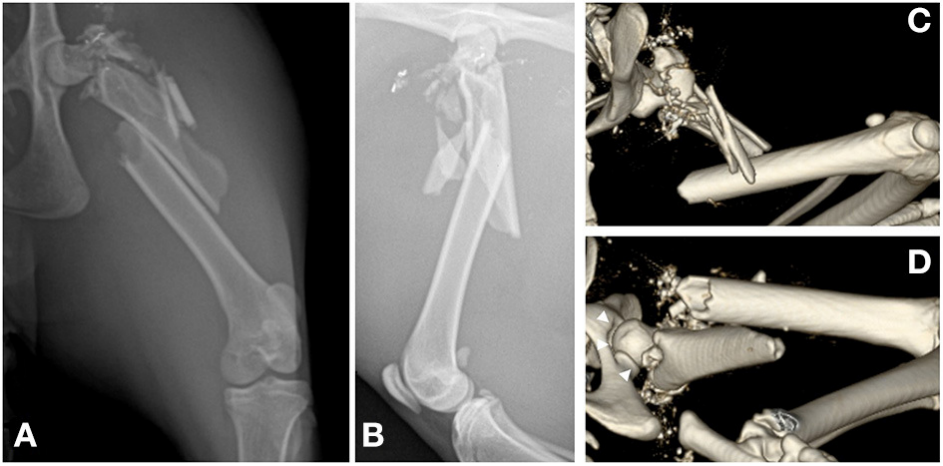
Preoperative radiographs and computed tomography scan of the left hind limb. **(A,B)** Craniocaudal **(A)** and mediolateral **(B)** radiographic views showing highly comminuted fractures at the proximal femur, including the neck, trochanteric, subtrochanteric, and the proximal half of the diaphysis. **(C,D)** Volume-rendered computed tomography scan of the cranial **(C)** and caudoventral **(D)** views showing the comminuted bones of the neck and trochanter and the comminuted spiral fracture of the proximal half of the femoral diaphysis. The hip joint is confirmed to be intact (white arrowheads).

A proximal segmental fracture of the left femur and multiple radiopaque objects of various sizes were identified on both plain radiographs and computed tomography scans (Figure 1). A comminuted spiral diaphyseal fracture and comminution of the neck and greater trochanter with an intact femoral head were identified on computed tomography. Injuries to the major organs were not identified. However, there was parenchymal damage to the left gluteal and thigh muscles.

The comminuted proximal femoral fracture was stabilized with a closed approach. Written informed consent for the surgical procedure and complications was obtained from the owner. The major bullet fragments embedded in the abdominal wall were removed. However, the other small intraabdominal bullet fragments were not removed due to the costs involved and since they did not pose any immediate clinical problems.

**Figure 2:**
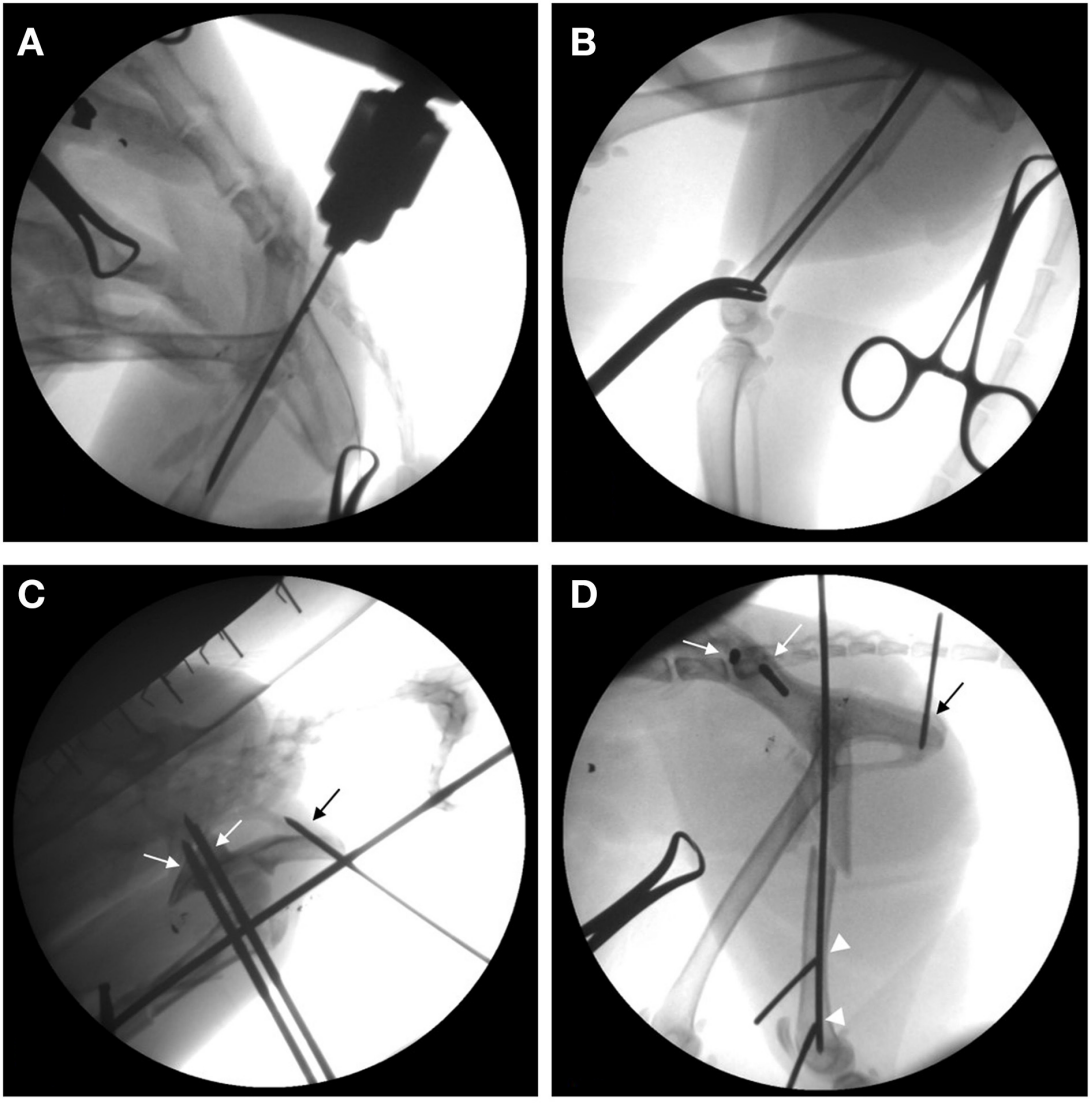
Intraoperative fluoroscopic series showing closed reduction and implant insertion in a 2-year-old cat with a comminuted proximal femur fracture. **(A,B)** The intramedullary pin is inserted into the trochanteric region, normograde from proximal to distal **(A)**, and it stabilizes the proximal and the distal femoral diaphyseal fragments **(B)**. **(C,D)** For trans-articular external skeletal fixation of the hip, three of the five positive-profile end-threaded pins are inserted to the left pelvis, including two pins in the ileum (white arrows) and the third pin in the ischium (black arrow), while the other two pins are placed in the distal fragments of the fractured femur (white arrowheads).

Under general anesthesia, the cat was placed in the right lateral recumbent position, and the left pelvic limb was prepared using a routine hanging limb preparation. Under fluoroscopic guidance, the femoral condyles were secured with a pair of AO forceps and indirect reduction was achieved by applying traction to the distal femur. A small skin incision was made medial to the greater trochanter, and a 2.4-mm Steinmann pin was inserted into the medullary canal of the femur in a normograde fashion (Figure 2A). The intramedullary pin occupied 47% of the medullary cavity at the isthmus (5.1 mm) and stabilized the major diaphyseal segments but not the comminuted femoral neck or the greater trochanter (Figure 2B).

**Figure 3:**
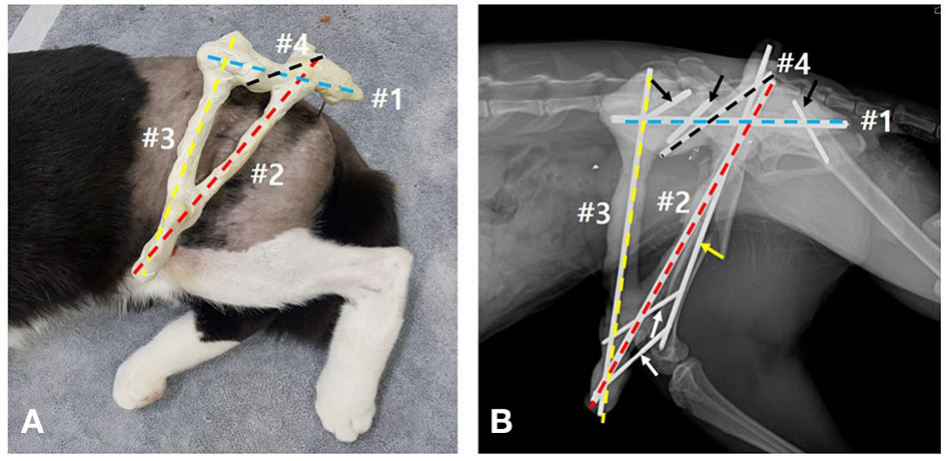
**(A,B)** Triangular frame composed of connecting bars with epoxy-putty molded pins for trans-articular external skeletal fixation of the hip: the 3.0-mm Steinmann pin (#1, blue dashed line) links the fixation pins placed in the left pelvis (black arrows); the other 3.0-mm Steinmann pin (#2, red dashed line) links to the fixation pins placed in the distal segment of the fractured femur (white arrows); #1 and #2 connecting bars are linked to each other at the intersection; the 2.4-mm Steinmann pin (#3, yellow dashed line) links to the #1 and #2 connecting bars; the other 2.4-mm Steinmann pin (#4, black dashed line) is tied into the intramedullary pin (yellow arrow) and links to the #1 connecting bar. Epoxy-putty covers all the pins composed of connecting bars including column of the pins and those connections.

To construct the TA-ESF of the hip, two 2.5 mm and one 1.8 mm positive-profile end-threaded pins were placed in the ilium and ischium, respectively. Then, one 2.0 mm and one 1.8 mm positive-profile end-threaded pins were also placed perpendicular to the distal femur (Figures 2C,D). The three pins placed in the ilium and ischium were connected to the first connecting rod, and the two pins in the distal femur were connected to the second connecting rod. The first and second connecting rods were linked to each other, and the third connecting rod was placed to link the first and second bars cranially. The preplaced intramedullary pin was tied into the first connecting rod. The connecting rods were fashioned using 3.0 mm Steinmann pins and epoxy resin (Figures 3A,B). For a stable triangular configuration, the magnitudes of forces and moments at intersection points were considered to distribute the external loads equally.

During this process, the limb was temporarily supported by an assistant to maintain appropriate rotation and angulation of the femur based on palpation of the condyles and the comminuted greater trochanter. This also allowed for fluoroscopic identification of the femoral head, femoral diaphysis, and linea aspera. Additionally, the coxofemoral joint was flexed and maintained at a normal weight-bearing angle while the epoxy resin polymerized. After polymerization, the left coxofemoral joint was fixed at ~90°. The fixator was lightly bandaged, and the cat was then re-positioned in left lateral recumbency to remove the bullet from the right abdominal wall.

**Figure 4:**
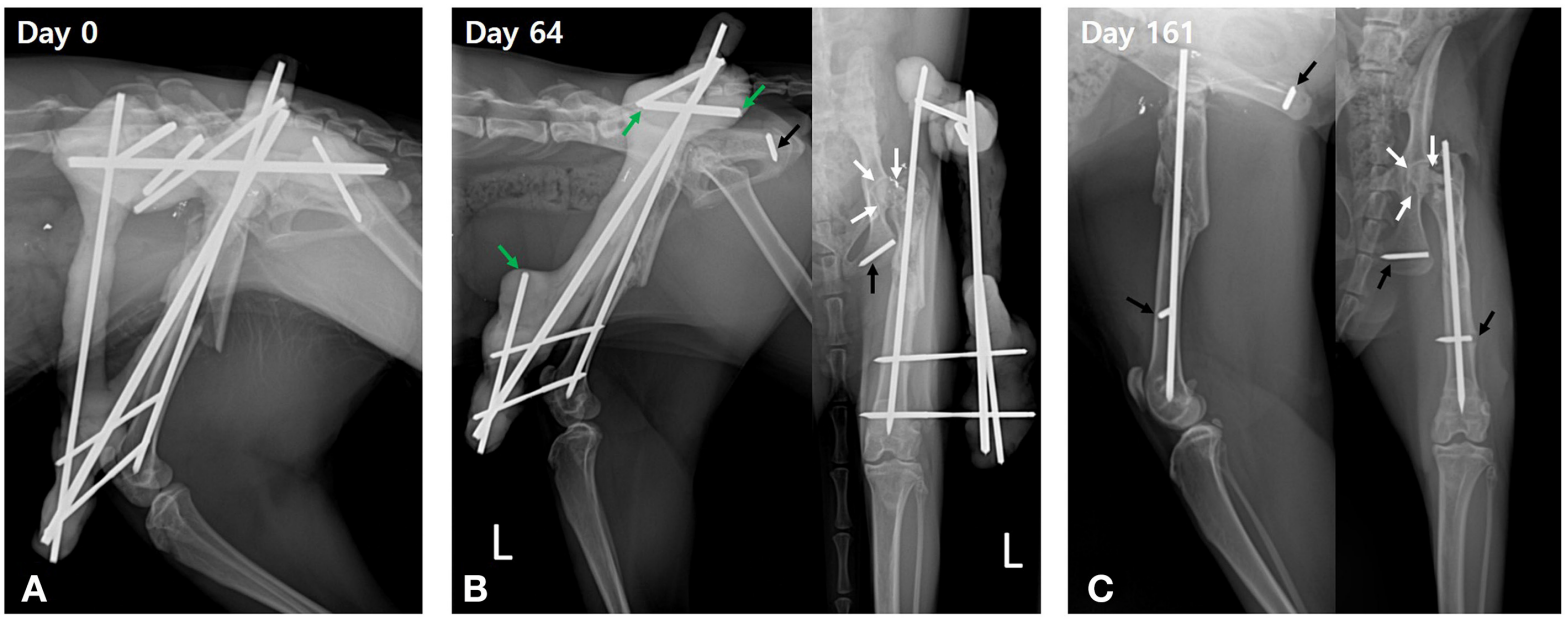
Postoperative serial radiographic views of the left femur. **(A)** Lateromedial radiograph acquired immediately after surgery shows good alignment and normal length of the left femur with trans-articular external skeletal fixation of the hip and a tied-in intramedullary pin. **(B)** On postoperative day 64, periosteal callus bridges the femoral fracture gap with cortical remodeling. Heterogenous opacity of the femoral neck and greater trochanter are identified on the craniocaudal radiographic view (white arrows). Trans-articular external skeletal fixation of the hip is disassembled to the linear tie-in frame by removal of fixation pins in the left pelvis and the associated connecting bars. The connecting bars are cut at the linkage area (green arrows) to preserve the linear tie-in frame. The fixation pin in the ischium is broken and partially embedded in the ischium (black arrow). **(C)** On day 161, the whole frame of the external skeletal fixation is removed, leaving the intramedullary pin and embedded part of the broken pins (black arrows). Fracture union showing good progression in bone healing is identified as a heterogeneous opacity with a mottled appearance at the femoral head and neck (white arrows).

The cat recovered from anesthesia and surgery without complications and was administered a constant-rate infusion of fentanyl for 24 h (fentanyl: loading, 4 μg/kg; constant-rate infusion, 4 μg/kg/h), followed by meloxicam for 4 days (0.1 mg/kg PO for 24 h) for analgesia. Postoperative radiographs were taken to verify appropriate placement of the pins and alignment of the femur (Figure 4A). Craniocaudal imaging was not possible due to the immovable left hip joint.

The day after surgery, the cat would not place weight on the left pelvic limb, which was very painful, and could not tolerate palpation of the left femur (Supplementary Video 1). On postoperative day 4, the cat was more comfortable, ambulatory, and could place nominal weight on the left pelvic limb. The cat was discharged 4 days after surgery with instructions to the owners to restrict the cat's activity, including confining the cat to a small room and taking it for short walks on a leash. The owners were also instructed to clean the fixation pin–skin interfaces daily with saline and chlorhexidine solution.

The cat was re-evaluated on postoperative days 18, 32, 46, 64, 80, 112, and 161. During these evaluations, the fixator was monitored for complications. Pain and limb function were subjectively assessed using an ordinal scoring system (Supplementary Table 1). Serial radiographs were also obtained to assess progression of the fracture toward union. By postoperative day 32, the cat had progressed to weight-bearing lameness. However, it still had a distinct head nod and mild pain on palpation of the left femur. On postoperative day 64, perceived pain and lameness were similar to those in the previous evaluation, and radiographs revealed an interfragmentary callus and cortical remodeling, bridging the diaphyseal femoral fracture gap. The femoral neck and greater trochanter were remodeling, exhibiting corticalization with heterogeneous opacity (Figure 4B). The fixation pin placed in the ischium had broken, and the triangular construct was partially disassembled to a linear tie-in construct by removing the fixation pins placed in the pelvis using Jacob's hand chuck. Additionally, the first and third connecting rods were cut near the area of linkage using a saw and pin cutter, while avoiding impairment of the remaining linear tie-in frame (Figure 4B). This disassembly re-established motion in the left coxofemoral joint. The cat showed more pain immediately after disassembly than before disassembly of the frame. However, it still placed weight on the left pelvic limb with notable lameness and a distinct head nod (Supplementary Video 2). Although, regained the range of motion of the left hip joint and passive manipulation, including flexion and extension of the hindlimb, were reduced, showing mild discomfort (Supplementary Video 3).

The cat's condition progressively improved over the next couple of weeks, and it did not appear to be in pain when examined on postoperative day 80. The examination on day 112 showed nearly normal gait (Supplementary Video 4). The range of motion of the left coxofemoral joint steadily improved and was nearly normal (40° of flexion and 140° of extension) by day 112. When evaluated on day 161, the cat did not have an appreciable lameness, no longer experienced pain on manipulation of the left pelvic limb, and could jump up onto an examination table. Radiographs demonstrated advanced remodeling of the comminuted greater trochanter and a callus bridging the diaphyseal fracture. Bone resorptions were identified in the femoral head and neck, represented by a mottled appearance of the head and thinning, sclerotic changes of the neck, and the rotational malalignment of the femoral head was confirmed as the head was superimposed over the femoral shaft (Figure 4C). The proximal fixation pin in the distal femur had broken. The remainder of the fixator was removed, leaving the intramedullary pin and broken segments of the fixation pins embedded in the bone (Figure 4C). The length of the left femur and the left pelvic limb muscle volume were comparable to those of the contralateral limb.

Telephone follow-up was conducted for 29 months postoperatively. The owner reported that the cat represented normal gait without any noticeable pain and lameness (Supplementary Video 5).

## Discussion

ESF, as chosen in this cat, is the preferred mode of trans-articular fixation in small animals for supporting tendon (4, 5) and ligament injuries (4, 6, 7). Further, it supports the primary implants for fractures (6, 8) of various joints, such as the stifle (4, 9–14), tarsal (5, 7, 10), and elbow (15). In particular, since the trans-articular fixation using ESF preserves the joint during the procedure (4–8), it is valuable for temporary immobilization of the associated joint without the articular invasion. Therefore, ESF was considered the most feasible option for temporary fixation of the coxofemoral joint in this cat, providing sufficient stabilization of the proximal femur, while preserving the coxofemoral joint. Additionally, with ESF, biological osteosynthesis, and ligamentotaxis can be enhanced through a closed approach. This is the biggest benefit of ESF since it preserves the fracture hematoma and periosteal vascularity while providing longitudinal distraction forces to hold the fragment closer together using surrounding soft tissues (16, 17).

The TA-ESF frame in the present study was creatively developed based on the anatomical features of the pelvis–distal femoral interconnection and physical distribution of the load. For stabilizing the comminuted proximal juxta-articular femur, the pelvis was utilized as the proximal segment instead of the comminuted proximal segment of the femur. The fixation pins in the pelvis were placed in the sacropelvic area of the ilium and the ischiatic tuberosity based on a previous study (18), considering stress concentration, amount of bony stock, and the risk of iatrogenic injury to the sciatic nerve. This TA-ESF frame of the hip was reinforced by making a triangular configuration, which placed a third connecting rod to induce load distribution. It is considered that this triangular configuration could reduce the load of the conventional TA-ESF frame, ultimately increasing the stability of the TA-ESF frame.

A previous case report revealed a similar TA-ESF frame of the hip in a cat (19). However, there are significant differences in the frame between the previous and present studies. Whereas, the previous frame secured the proximal femoral segment, the frame of this study was a more complex fracture that could not secure the proximal femoral segment due to its high comminution. Thus, the previous TA-ESF frame of the hip only served to support the primary ESF placed in the femur, while the TA-ESF frame of the hip in the present study played a primary role in stabilizing the comminuted proximal juxta-articular femur in the pelvis utilized as the proximal segment instead of the comminuted proximal segment of the femur. Further, to restore the femoral length and to enhance the bending resistance, the tied-in configuration, which was not used in the previous frame, was additionally constructed in the present study.

Femoral malalignment and deformities were the major concerns for this patient. To align the fractured femur and avoid postoperative deformities, the anatomical landmarks, including the greater trochanter, femoral condyle, and linea aspera, were consistently assessed by palpation and fluoroscopic guidance. The greater trochanter, though highly comminuted, could be easily palpated since its fragments were not scattered and remained closely enough to maintain a near-normal shape. Thus, it could be used as a landmark to evaluate the rotational alignment of the femur with the condyles and the linea aspera. Using these landmarks, intramedullary pinning was performed to restore the length and axial alignment of the femur, thus, helping the cat gain functional recovery of the injured gait. However, due to the limitations of the closed approach in this kind of comminuted fracture for bridging and biological osteosynthesis, each bone fragment could not be opposed accurately, and this might lead to delayed bone healing and the residual rotational malalignment of femoral head. Additionally, since the femoral neck had a highly comminuted fracture, the anteversion and inclination angle of the femoral neck could not be accurately assessed perioperatively. Therefore, the appropriate angles were estimated intraoperatively under fluoroscopic guidance by comparing the locations of the center of the femoral head, greater trochanter, and ideal axis of the shaft to avoid increasing these angles markedly. If an abnormal anteversion and inclination angle occurred despite these preventive efforts, a salvage procedure was planned, involving femoral head and neck osteotomy.

Another major concern was postoperative degenerative changes of the femoral head, possibly due to immobilization of the hip joint and ischemic osteonecrosis secondary to reduced blood supply. Joint immobilization is the most significant cause of degenerative joint disease associated with trans-articular ESF (11, 15, 20), but it was not considered the primary cause of femoral head degeneration in this cat. Previous experimental and clinical studies have reported that prolonged joint immobilization alters the biochemical characteristics of synovial joints (3, 20), inducing softening of the articular cartilage and decreasing cartilage thickness, leading to the development of degenerative joint disease (10, 14). These changes were apparent after 11 weeks of immobilization. However, they were reversible even after 11 weeks of immobilization. Thus, no significant difference was seen between the immobilized joint and the age-matched controls after a remobilization period of 50 weeks (21). Additionally, weight-bearing during immobilization of the limb might promote recovery of articular cartilage (13). In this cat, the left hip joint was immobilized for 64 days, which was not long enough to induce degenerative changes in the articular cartilage, relative to the immobilization period of previous studies (21). Moreover, consistent weight bearing-use of the limb during the immobilization period may have helped minimize these changes and facilitated recovery of the articular cartilage. Therefore, it is less likely that joint immobilization would have contributed significantly to degenerative changes of the affected femoral head in this cat.

Damaged blood supply to the proximal femur was considered to be the main cause of degenerative changes in the femoral head of this cat. Arterial branches that supply blood to the femoral head propagate from extra osseous vessels, such as the lateral and medial circumflex femoral arteries and the caudal and cranial gluteal and iliolumbar arteries, which are supplied from the surrounding soft tissue to the femoral head through the femoral neck and trochanteric fossa (22, 23). Compromise of this vascular network may induce changes in the femoral head and neck, such as resorption and degenerative joint disease (24–26). Although, avascular necrosis of the femoral head was not a common sequela in a dog and cat with femoral head and neck fractures (25), the comminuted neck, trochanter, and the surrounding soft tissue injury from the gunshot in the present case may have caused more extensive compromise of this vascular network than in cases with only neck fracture, markedly disrupting the blood supply of the femoral head and neck, and potentially led to the development of avascular necrosis in this cat. Additionally, the femoral neck was not stabilized in this cat because the comminuted neck fragments could not hold the implants engaging the neck. Thus, this consistent instability may also contribute to sclerotic changes and non-union of the neck and degenerative changes of the femoral head (25). However, it did not become a serious clinical problem causing pain and lameness since the cat is currently very active with a normal gait, 29 months after surgery.

At the last follow-up, lack of bone healing was identified in the proximal diaphyseal segments of the radiograph, despite functional recovery of the injured leg. This is thought to be due to the characteristics of a comminuted fracture and impaired blood supply caused by high energy-induced trauma, such as gunshot injury. Further, the sustained instability of the proximal segment caused by the inability to place implants in the comminuted proximal segment was also considered to have contributed to delayed bone healing. To increase the stability of the proximal segment, the preferred surgeries for biologic osteosynthesis of comminuted fractures, such as minimally invasive plate osteosynthesis or an interlocking nail, were considered. However, these conventional methods were unsuitable for this cat since the proximal segment was comminuted and not durable enough to place the implants. And, the femoral head and neck osteotomy with repair of diaphyseal fracture were also considered as a salvage procedure, however, since the femoral head was intact, it was decided to withhold and considered as a later option. Due to the limitations of conventional surgery, a TA-ESF of the hip was used for bridging osteosynthesis and a triangular configuration was developed to reduce the load and increase the stability of the TA-ESF. Nevertheless, since a secure connection between TA-ESF and the proximal segment was not possible due to the comminuted and longitudinally fractured proximal diaphyseal fragment, it might have led to constant instability of the proximal segment inducing deficient bone healing.

Performed surgery and contributed to the conception and design There are some limitations to the present case report. Although, this cat had several problems requiring long-term follow-up, this was not possible. In the radiograph at the last follow-up visit, lack of fracture healing, degenerative change of the affected femoral head and thinning and sclerotic changes of the femoral neck were identified. These abnormalities should have been consistently monitored because they might progress to malunion of the fractured femur, especially non-union of the femoral neck and degenerative joint disease of the hip joint. Moreover, the intramedullary pin, which was left due to insufficient remodeling of the femoral diaphysis, was to be removed as soon as complete union was confirmed. For this reason, constant long-term follow-up was strongly recommended to the owner. However, the owner did not presented after ESF removal, since the cat could walk normally, without any signs and the owner had financial and long-distance restrictions. Additionally, partial failure of this TA-ESF of the hip due to several weak points, including fracture of some fixation pins and instability of the proximal segment, were seen in this case. Although, these problems did not affect functional recovery in this cat, further, investigation on the number and location of taxation pins and frame of connecting bars with more cases is necessary to improve the stability and configuration of this TA-EFS of the hip.

## Concluding Remarks

In summary, despite the lack of fracture healing, TA-ESF provided a satisfactory clinical outcome, including successful biological osteosynthesis and functional recovery of a comminuted fracture of the proximal femur caused by gunshot injury in this cat. The ESF configuration was determined based on the principle of physical force distribution, and the tied-in configuration enhanced its stability. Further, the use of epoxy made it possible to construct this complicated configuration easily without the limitations of angles, planes, or the position of fixation pins. This configuration is a feasible surgical option for such highly comminuted juxta-articular fractures of the proximal femur, in which the implant cannot be secured to the proximal segment.

## Data Availability Statement

The original contributions presented in the study are included in the article/Supplementary Material, further inquiries can be directed to the corresponding author/s.

## Ethics Statement

Ethical review and approval were not required for the animal study because this is a case report which has been written using clinical data. Written informed consent was obtained from the owners for the participation of their animal in this study.

## Author Contributions

H-JH performed surgery and contributed to the conception and design of the work, analyses of the data, and writing the manuscript. KL contributed to providing the concept of the most stable frame for TA-ESF based on physics theory and preparing the relevant part of manuscript, while H-YY supervised aftercare, and critically revised the manuscript. All authors contributed substantially to the study design, preparation, and final approval of the manuscript.

## Conflict of Interest

The authors declare that the research was conducted in the absence of any commercial or financial relationships that could be construed as a potential conflict of interest.

## Abbreviations

ESF: External Skeletal Fixation
TA-ESF: Trans-articular External Skeletal Fixation.
