# Supplementary material for: Case Report: Trans-Articular External Skeletal Fixation of the Hip for a Highly Comminuted Juxta-Articular Fracture of the Proximal Femur Caused by Gunshot Injury in a Cat
Source: Front Vet Sci. 2021 Jul 19;8:652847. doi: 10.3389/fvets.2021.652847 (PMC8328486; doi:10.3389/fvets.2021.652847)
Supplement: Supplementary file 1 [file Table_1.docx]

Supplementary Material

Supplementary materials are total 6 files including 1 table and 5 videos (MP4).

**Supplementary Table 1**. Postoperative visual observation of pain and gait in the cat based on the subjective scoring system.

|  | **Scoring** | **Postoperative day** | | | | | | | | |
| --- | --- | --- | --- | --- | --- | --- | --- | --- | --- | --- |
|  |  | 0 | 4 | 18 | 32 | 46 | 64 | 80 | 112 | 161 |
| **Pain** | **1**: no pain elicited on palpation of affected joint |  |  |  |  |  |  | ■ | ■ | ■ |
|  | **2**: mild pain elicited, e.g., turns head in recognition |  |  |  | ■ | ■ |  |  |  |  |
|  | **3**: moderate pain elicited, e.g., pulls limb away |  | ■ | ■ |  |  | ■ |  |  |  |
|  | **4**: severe pain elicited, e.g. vocalizes or becomes aggressive |  |  |  |  |  |  |  |  |  |
|  | **5**: will not allow examiner to palpate joint due to pain | ■ |  |  |  |  |  |  |  |  |
| **Gait** | **0**: Normal |  |  |  |  |  |  |  |  | ■ |
|  | **1**: Sound walking, but weight shifting and mild lameness noted at a trot |  |  |  |  |  |  |  | ■ |  |
|  | **2**: Mild weight-bearing lameness noted with the trained eye |  |  |  |  | ■ |  | ■ |  |  |
|  | **3**: Weight-bearing lameness, typically with a distinct head nod |  |  |  | ■ |  | ■ |  |  |  |
|  | **4**: Significant weight-bearing lameness |  |  | ■ |  |  |  |  |  |  |
|  | **5**: Toe-touching lameness |  | ■ |  |  |  |  |  |  |  |
|  | **6**: Non-weight-bearing lameness | ■ |  |  |  |  |  |  |  |  |

**Supplementary Video 1.** Gait status on postoperative day 1.

**Supplementary Video 2.** Gait status on postoperative day 64.

**Supplementary Video 3.** The range of motion of the left hip joint after the frame disassembly on postoperative day 64.

**Supplementary Video 4.** Gait status on postoperative day 112.

**Supplementary Video 5.** Gait status on postoperative months 29.
